# Detection of periodontal bone loss and periodontitis from 2D dental radiographs via machine learning and deep learning: systematic review employing APPRAISE-AI and meta-analysis

**DOI:** 10.1093/dmfr/twae070

**Published:** 2024-12-05

**Authors:** Yahia H Khubrani, David Thomas, Paddy J Slator, Richard D White, Damian J J Farnell

**Affiliations:** School of Dentistry, Cardiff University, The Annexe, University Dental Hospital, Heath Park, Cardiff CF14 4XY, United Kingdom; School of Dentistry, Jazan University, Jazan 82817, Saudi Arabia; School of Dentistry, Cardiff University, The Annexe, University Dental Hospital, Heath Park, Cardiff CF14 4XY, United Kingdom; School of Computer Science and Informatics, Cardiff University, Cardiff CF24 4AG, United Kingdom; Cardiff University Brain Research Imaging Centre, School of Psychology, Cardiff University, Cardiff CF24 4HQ, United Kingdom; Department of Clinical Radiology, University Hospital of Wales, Cardiff CF14 4XW, United Kingdom; School of Dentistry, Cardiff University, The Annexe, University Dental Hospital, Heath Park, Cardiff CF14 4XY, United Kingdom

**Keywords:** artificial intelligence, deep learning, panoramic radiographs, periapical radiographs, periodontitis

## Abstract

**Objectives:**

Periodontitis is a serious periodontal infection that damages the soft tissues and bone around teeth and is linked to systemic conditions. Accurate diagnosis and staging, complemented by radiographic evaluation, are vital. This systematic review (PROSPERO ID: CRD42023480552) explores artificial intelligence (AI) applications in assessing alveolar bone loss and periodontitis on dental panoramic and periapical radiographs

**Methods:**

Five databases (Medline, Embase, Scopus, Web of Science, and Cochrane’s Library) were searched from January 1990 to January 2024. Keywords related to “artificial intelligence”, “Periodontal bone loss/Periodontitis”, and “Dental radiographs” were used. Risk of bias and quality assessment of included papers were performed according to the APPRAISE-AI Tool for Quantitative Evaluation of AI Studies for Clinical Decision Support. Meta analysis was carried out via the “metaprop” command in R V3.6.1.

**Results:**

Thirty articles were included in the review, where 10 papers were eligible for meta-analysis. Based on quality scores from the APPRAISE-AI critical appraisal tool of the 30 papers, 1 (3.3%) were of very low quality (score < 40), 3 (10.0%) were of low quality (40 ≤ score < 50), 19 (63.3%) were of intermediate quality (50 ≤ score < 60), and 7 (23.3%) were of high quality (60 ≤ score < 80). No papers were of very high quality (score ≥ 80). Meta-analysis indicated that model performance was generally good, eg, sensitivity 87% (95% CI, 80%-93%), specificity 76% (95% CI, 69%-81%), and accuracy 84% (95% CI, 75%-91%).

**Conclusion:**

Deep learning shows much promise in evaluating periodontal bone levels, although there was some variation in performance. AI studies can lack transparency and reporting standards could be improved. Our systematic review critically assesses the application of deep learning models in detecting alveolar bone loss on dental radiographs using the APPRAISE-AI tool, highlighting their efficacy and identifying areas for improvement, thus advancing the practice of clinical radiology.

## Introduction

### Rationale

Periodontitis is a serious multifactorial periodontal infection that damages the soft tissues and bone around teeth and is linked to systemic conditions.^1^ The prevalence of periodontitis is estimated to be about 50% in the United States based on the American Academy of Periodontology,^2^ and around 10%-15% globally suffer from severe cases that cause loss of teeth.^2^ Periodontitis is a complex multifactorial process that is initiated with bacterial plaque accumulation, and biofilms, followed by a host immune reaction or inflammatory response.^1^^,^^3^ If not treated, periodontitis progression will eventually lead to teeth loss and impaired oral function.^4^ Although no conclusive cause-and-effect has been established, studies have correlated periodontitis as a possible predisposition for systematic conditions, such as cardiovascular diseases, respiratory tract infections, adverse pregnancy outcomes, Alzheimer’s disease, and oral and colorectal cancer.^4^^,^^5^

In addition to clinical findings of periodontal disease, dental radiography is an integral part of diagnosis and treatment planning as it provides a comprehensive evaluation of hard dento-alveolar structures, as well as calculus depositions, shape of roots, and alveolar bone level.^6–8^ Several radiographic techniques are used for periodontal examination. Bitewing provides limited details on the maxillary and mandibular teeth crowns and the alveolar crest level. Full mouth series of parallel periapical radiographs have been considered “the gold standard” for periodontal evaluation. This is because periapical radiographs provide information on teeth and supporting structures with relatively low-dose radiation, while still providing images of high resolution and that are of good quality.

Panoramic radiographs have become the most commonly used modality in dental examination and periodontal evaluation. Such radiographs provide a comprehensive view of the maxillofacial structures. They also capture maxillary and mandibular teeth and the alveolar bone in one image and in a few seconds. Furthermore, they involve a relatively low radiation dose and yet still give acceptable image quality.^6^^,^^9^ The introduction of cone beam computed tomography (CBCT) allowed the 3D evaluation of periodontal structures and a comprehensive evaluation of periodontal defects such as furcation defects, fenestration, and dehiscence and postsurgical evaluation of regenerative periodontal procedures.^10^^,^^11^ However, CBCT can lead to a high dose of radiation, as well as inherent artefacts, and so should not be used in routine examination procedures.^12^^,^^13^

Currently there is a surge in artificial intelligence (AI) applied to all aspects of dentistry, with a wide range of applications ranging from simple task management to complicated diagnostic evaluation and tools in decision making.^14^ A recent innovation has been the introduction of deep learning (DL), which is a form of AI that often involves the use of neural networks. Some dental/oral health examples in the literature are cancer cell detection and healing evaluation, enhanced restoration margins adaptation, caries detection and shade selection, pre-and post-orthognathic surgical evaluation, cephalometric analysis, periodontal evaluation, and bone loss detection, CAD-CAM and 3D printing for implant treatment, root canal morphology, canal length, and vertical root fracture.^14^^,^^15^ Additionally, Hunge *et al*^16^ highlighted AI applications in 3D diagnostic imaging in their narrative review, especially relating to multidetector CT and CBCT to discover and delineate jaw cysts and tumors, lymph node metastases, salivary glands diseases, temporo-mandibular joints (TMJs), maxillary sinuses, mandibular fracture, and dento-maxillofacial deformities. DL is extensively utilized for segmentation tasks,^17^^,^^18^ accurately delineating structures in dental radiographs.^19^ Additionally, DL models are applied for both segmentation and classification, enabling the precise identification and categorization of dental conditions.^20–22^

### Objectives

Two systematic reviews^23^^,^^24^ have explored the application of DL in evaluating periodontal bone loss assessed via dental radiographs, excluding CBCT. There has been a noticeable increase in the number of publications since these systematic reviews were published. These papers addressed the different DL models and may provide additional information important in improving the diagnostic accuracy of these models and help in clinical implementation and support in the decision-making process. Therefore, this systematic review aims to explore, analyze, and summarize the application of DL models in evaluating periodontal bone loss using the newly developed APPRAISE-AI Tool for Quantitative Evaluation of AI Studies for Clinical Decision Support.^25^

## Methods

### Eligibility criteria

The articles were collected in January 2024 following the PICO/PIRO (Population, Intervention/Index Test [AI-Model], Comparison/Reference Standard, and Outcome) question format was followed during the search, P: Patient with periodontal bone loss/periodontitis; I: Radiographic image evaluation with AI; C: Radiographic evaluation of clinician or relative to an established ground truth and/or gold standard, which according to authors of the papers was established either from patients records or experts who pre-labelled the images; O: periodontal bone loss detection and classification accuracy. We used PICO/PIRO instead of PICOS as almost all studies included did not discuss the study design and all seemed cross-sectional. This highlights common problems related to transparency and reporting that are later discussed in the results and discussion section. This study included all articles published from January 1990 to January 2024; papers that were written in English; articles that applied any type of AI models, such as RCNN or SVM, to evaluate the periodontal bone level, periodontitis, and/or periodontal diseases on intraoral or extraoral radiographs, such as Periapical, Bitewings, and Panoramic radiographs. Although SVM is a machine learning (ML) algorithm, it was retained due to relatively little evidence found in the literature. It fits both inclusion criteria and keywords used in the search. We also noted that it has been used as a comparison model in some of the DL studies we included.^26^^,^^27^ We excluded studies published before 1990, non-English articles, and conference abstracts without fully published documents.

### Information sources

The systematic review has been registered with PROSPERO (ID: CRD42023480552). Search parameters and keywords were developed by the authors. Keywords were set initially by using the Population, Intervention/Index Test (AI-Model), Comparison/Reference Standard, and Outcome (PICO/PIRO) approach. These keywords were then iterated between all authors until a mutually agreed set of terms was found.

### Search strategy

The databases searched in January 2024 were Medline via Ovid (13 papers), Embase via Ovid (41 papers), Scopus through Elsevier (83 papers), Web of Science (62 papers), and Cochrane Library (5 papers). The total initial search yielded 204 that were exported to the EndNote Library. Terms used: (“Artificial intelligence” OR “AI” OR “Machine learning” OR “Neural network” OR “Deep Learning” OR “Convolutional neural networks”) AND (“Alveolar bone loss” OR “Periodontal bone loss” OR “Periodontal disease” OR “Periodontitis” OR “Diagnosis of periodontal bone loss” OR “Detect alveolar bone loss”) AND (Radiograph OR “Dental radiograph” OR “Periapical radiograph” OR “Panoramic radiograph” OR “Radiographic imaging” OR “Cone beam computed tomography” OR “CBCT”).

**Figure 1.**
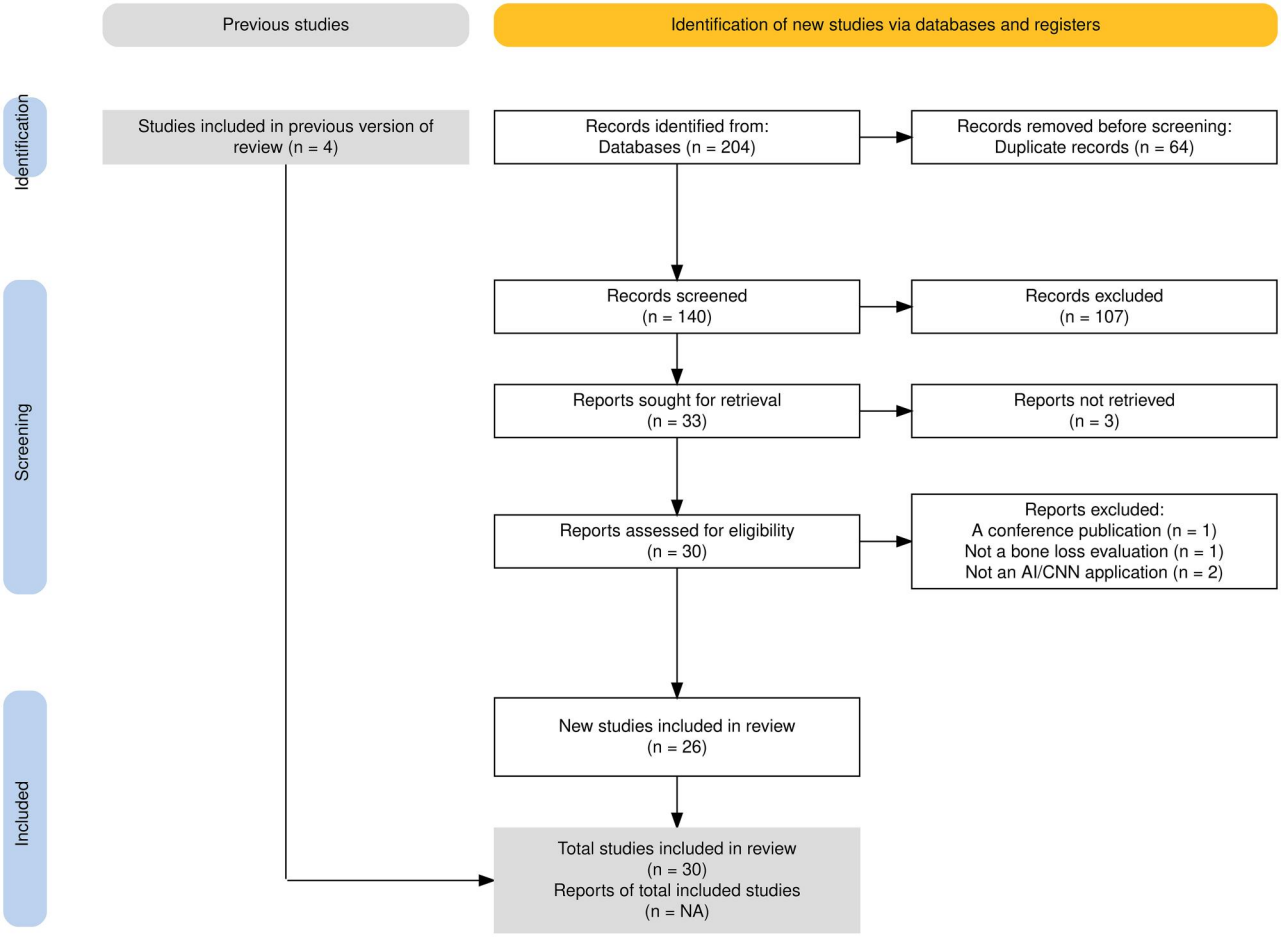
PRISMA flowchart for the study.

The selection process, data extraction, analysis, and reporting procedures in this review follow the PRISMA (Preferred Reporting Items for Systematic Reviews and Meta-Analysis) guidelines.^28^ A PRISMA chart^29^ shown in Figure 1 illustrates the stages of data extraction. The database search yielded 204 papers, 64 of which were removed during the removal of duplicate sources. An additional 107 articles were found to be irrelevant according to our exclusion criteria and were removed during initial screening. Thirty-three articles were included in the database search. Additionally, 4 papers not found in the database search were retrieved from the previous systematic reviews^23^^,^^24^ and included in the study.

### Data collection

Those 37 articles were uploaded to the Rayyan systematic review collaboration website to be evaluated by all other researchers. Two researchers critically appraised these articles. Three full article texts could not be retrieved and were removed from the study. Four papers were also excluded during the final evaluation as they did not meet the selection criteria as shown in the PRISMA flowchart. Finally, 30 eligible articles, on which all reviewers agreed, were included in this systematic review. Eleven of these 30 articles provided common outcomes that could be used in meta-analyses.

### Risk of bias assessment

Critical appraisal of the final 30 papers was performed by 2 independent reviewers to reduce the risk of bias. The appraisal tool used in this study was published by Kwong et al^25^ (APPRAISE-AI Tool for Quantitative Evaluation of AI Studies for Clinical Decision Support). Each paper was scored independently using the APPRAISE-AI tool by 2 independent reviewers (2 of the authors) on the scale going from a minimum of 0 (extremely poor quality) to 100 (extremely good quality), according to APPRAISE-AI.^25^ The 2 raters agreed within 6 points for 25 out of 30 papers, which indicates good agreement between the 2 raters in absolute terms (with respect to overall scores in the range [0,100]), and a composite mark (ie, the mean of the 2 scores) was used as the final score for each paper in this case. For those cases where initial disagreement was greater than 6 marks, the 2 reviewers re-evaluated each paper and agreed on a final score mutually. Thus, a robust estimate of quality was determined via the APPRAISE-AI score.

Six domains were identified for the APPRAISE-AI critical appraisal tool [25]: clinical relevance (maximum domain score = 4), data quality (maximum domain score = 24), methodological conduct (maximum domain score = 20), robustness of results (maximum domain score = 20), reporting quality (maximum domain score = 12), and reproducibility (maximum domain score = 20). Scores for each of these domains could be determined readily also and they were found to be extremely useful in analyzing the strengths and weaknesses of the article used here. In order to facilitate comparison between these domain scores, they were also scaled linearly in the range minimum of 0 (extremely poor quality) to 100 (extremely good quality). The specific questions used for critical appraisal via the APPRAISE-AI tool are presented in the Supplementary Material to this article.

### Effect measures

The performance of DL models in detecting periodontal bone loss/periodontitis was measured with different parameters across studies. The following performance measures were used: accuracy, sensitivity (recall), specificity, precision (positive predictive value, PPV), F1-scores, negative predictive value (NPV), and the area under the receiving characteristic curve (AUC/ROC). Segmentation of features and localization accuracy were evaluated with intercession over union (IoU), dice similarity coefficient (DSC), Jaccard Index (JI), pixel accuracy (PA), and mean average precision (mAP). Note however that there was not enough data for meta analysis to be carried out for segmentation tasks. Bone loss or periodontitis was generally measured on a binary scale (no bone loss or bone loss). However, some studies^22^^,^^30^ had an ordinal scale and this is dichotomized by us explicitly here to form a binary scale (eg, no bone loss or *any* bone loss).

### Syntheses methods

#### Meta-analysis

Measures such as sensitivity, specificity, accuracy, PPV, NPV, and even F1-scores (etc.) are all ratios of 2 integers, where the numerator counts the number of “events” with respect to some (perhaps effective for F1-scores) sample size (ie, the denominator). (Note also that the units of sampling were images rather than subjects in all papers.) Each of these measures lies in the range [0,1] and (crucially) values for these measures have a common meaning across all articles, ie, values near to 0 indicate extremely poor performance and values near to 1 indicate extremely good performance. Thus, we can treat each measure as a simple proportion, and standard methods of meta-analysis for a proportion can be employed. (Note that there were no consistent control groups (or methods) and so meta-analyses via odds ratios or relative risks could not be carried out.) Here, pooled point estimates and 95% confidence intervals of a single proportion via meta-analysis were found using the “metaprop” command for the statistical software package “meta” in the statistical software environment R V3.6.1. The default “arcsine” transformation was used here to calculate an overall proportion, although other transformations (eg, logit, double arcsine, logarithm, etc) all gave similar results (this was tested explicitly in all cases), which is an excellent test of the method.

Note that some papers used multiple types of neural networks and results are quoted here for each type of network. Augmented data were used in some papers, which inflated the effective sample size by multiple orders of magnitude compared to the other studies and so strongly affected confidence intervals. Subgroup meta-analyses of augmented versus no-augmentation is carried out here in addition to overall meta-analyses including results from all papers. Funnel plots and statistical tests of bias did not indicate that bias was strong, where there were enough papers to allow this analysis to be carried out for all performance measures. Note finally that random-effects meta-analysis was carried out for those cases with larger amounts of heterogeneity (ie, *I*^2^ values greater than approximately 50% and *P *<* *.05 for tests of heterogeneity). Sensitivity analyses were carried out where obvious outliers were detected, ie, meta-analysis was repeated with any outliers removed and results were compared to the original analysis containing all studies.

## Results

### Study selection and study characteristics

Table 1 presents the evidence table for all 30 articles based on authors, year of publication, country, sample size and type of images, AI software, main findings, statistical analysis, and appraisal score. The results show an increase in the number of publications in the year 2023 compared to previous years, with 11 out of 30 papers published in 2023,^3^^,^^4^^,^^9^^,^^26^^,^^31–37^ 8 papers in 2022,^22^^,^^30^^,^^38–43^ 4 papers in 2021,^20^^,^^44^^,^^49^^,^^50^ 4 papers in 2020,^27^^,^^45^^,^^51^^,^^52^ 2 papers in 2019,^46^^,^^47^ and only one paper in 2018.^48^

**Table 1.** Summary of all articles included in the systematic review.

Authors year, country	Sample size and type	AI-software	Main study findings	Statistical analysis	Appraisal score of 100
Vollmer et al 2023,^3^ Germany	1755 panoramic radiographs	Key points RCNN (ResNet-50-FPN)	Ground truth: An expert drew the bounding box and determined the key points. The model showed low accuracy in keypoints and bounding box detections.	**Key points:** Precision 0.632 Recall 0.579 **Bounding box:** precision 0.758	53.5
Saylan et al 2023,^4^ Turkey	1543 panoramic radiographs	YOLO-v5x	Ground truth: Two specialists labelled the images. The model achieved good results in detecting alveolar bone loss (ABL). Regional segmentations produce better results compared to total detection. Maxilla results are better than mandible.	Recall 0.75 Precision 0.76 F1-score 0.76	57
Sameer et al 2023,^31^ India	116 panoramic radiographs	Support vector machine (SVM) and random forest (RF)	Ground truth: Three dentists manually segment the images. RF results were more accurate compared to SVM model in binary classification of alveolar bone loss. RF outperformed SVM in bone loss classification	Accuracy 0.83 Recall 0.92 Specificity 0.67 Negativity values 0.75	32.5
Ryu et al 2023,^32^ Korea	4083 panoramic radiographs	Faster R-CNN	Ground truth: Two dentists labelled the images with the bounding box and annotated the periodontal condition. Additionally, a comparison between the models and 2 dentists was performed. The model showed high accuracy and reproducibility results in detecting periodontally compromised teeth (PCT). The model showed comparable results to trained dentists with inter- and intra-examiners correlation (ICC) of 0.94. The regional grouping produced better results. The software was able to differentiate dentate from edentulous areas and detect PCT regardless tooth positions.	Precision 0.90 Recall 0.90 F1-socre 0.90 AUC 0.91 Overall averages of all indices: PCT 0.84 Healthy 0.88	52.5
Mao et al 2023,^33^ Taiwan	300 periapical radiographs	GoogLeNet, InceptionV3 Vgg19 AlexNet	Ground truth: Clinical and radiographic diagnosis was already established in patient files for the included images. Deep learning models detected Furcation involvement with high accuracy of about 95%. Images preprocessing methods, such as segmentation and masking techniques, are crucial and improved CNN models performance; hence post-processing results are better. The model detected single and multi-rooted teeth with high accuracy, about 97%.	**Accuracy: 95%** GoogleNet 0.95 Inception 0.94 Vgg19 0.92 AlexnNet 0.95 **Recalls:** GoogleNet 0.96 Inception 0.85 Vgg19 0.74 AlexnNet 0.87 **Precision**: GoogleNet 0.92 Inception 0.83 Vgg19 0.68 AlexnNet 0.80	46.5
Liu et al 2023,^34^ China	2275 panoramic radiographs	Alexnet	Ground truth: Three periodontists assigned and correlated the radiographic diagnosis with patient files. Additional comparison between the model and 6 clinicians. The model trained and tested on panoramic radiographs from 2 hospitals produced high accuracy results comparable to 3 periodontists and significantly superior to 3 general dentists. The model has significantly faster reading time than clinicians. Significant correlation between CNN score and severity of periodontitis (Spearman 0.8, *P* < .05) No significant effect of confounders such as coronal restoration on alveolar bone loss detection.	**Accuracy:** PAR-CNN 0.80, Periodontists’ 0.81 Dentists 0.69 **Sensitivity/Recall:** PAR-CNN 0.82 Periodontists’ 0.827 Dentists 0.70 **Specificity:** PAR-CNN 0.78, Periodontists 0.80, Dentists 0.673 **Mean time in second,** PAR-CNN 0.027 Periodontists’ 6.042 Dentists 13.105 **PAR-CNN AUC:** First test = 0.84 and in second test 0.79	70.5
Kong et al 2023,^35^ China	1747 panoramic radiographs	2-stage PDCNN	Ground truth: Three professionals labelled the images. PDCNN achieved high detection and classification accuracy of radiographic bone loss (RBL) outperforming existing model, YOLO-v4, and Faster R-CNN. Despite being 2-stage, PDCNN speed was comparable to 1-stage networks. Model performance factors, such as loss of function, feature maps, and connection modules affect model performance and accuracy.	**Accuracy (ACC):** PDCNN: 0.762. Faster R-CNN: 0.727. Centernet Hourglass: 0.707. Centernet ResNet-50: 0.659. YOLO-v4: 0.724. RetinaNet: 0.701.	59.5
Karacaoglu et al 2023,^26^ Turkey	87 periapical of dry mandible	**Radiomedics web-based platform**: KNN, SVM, XGBoost, RF, LR, and DT.	Ground truth: The periodontal defects were created manually prior to radiographic examinations. AI models were compared to an expert. Human observer and machine learning (ML) ability to detect periodontal defects were significantly different from the gold standard results. However, no significant difference between human and ML. Overall results of the modifiers in this model are acceptable but show variations and ranges from 0.5 to almost 0.9.	**KNN**: Precision 0.67, Recall 0.50, F1-score 0.57. **SVM, XGBoost, RF, and LR:** Precision 0.80, Recall 1, F1-score 0.89. **DT:** Precision 0, Recall 0, F1-score 0.	54
Chen et al 2023,^36^ Taiwan	336 periapical radiographs (PA)	U-Net and Mask-RCNN	Mask-RCNN for teeth segmentation and U-Net for tissue calcifications. Ground truth: Three periodontists annotated the images. The model showed high accuracy and reliability in detecting alveolar bone loss and staging periodontitis on PA compared to 3 periodontists with high Pearson’s correlation coefficient (PCC) and interclass correlation coefficient (ICC) values. It tends to stage toward severe side of the disease, with highest accuracy for stage III. It showed lower misdiagnosis cases evident by high sensitivity and NPP. Model accuracy enhanced by post-processing and augmentation.	**CNN vs. periodontists**: PCC = 0.828 (*P* < .01). ICC = 0.765. **CNN performance:** AUROC 0.946, F1 = 0.841, Recall = 0.97, Specificity = 0.638, PPV = 0.742, NPV = 0.952. **Stages accuracy:** I = 64%, II = 74%, & III = 94%. **Periodontists’ performance:** AUROC = 0.964, F1 = 0.891, Sensitivity/recall = 0.88, Specificity = 0.906, PPV = 0.915, NPV = 0.889.	52
Chen et al 2023,^37^ Taiwan	8000 periapical radiographs	YOLOv5	Ground truth: Five senior dentists annotated the images. The model detected teeth position and shape, and interproximal alveolar bone loss with high accuracy. The model accuracy was comparable to dentists. The authors mentioned comparison with dentists; However, no values were provided.	**Accuracy:** Teeth position 88.8% Teeth shape and segmentation 86.3% Bone level and segmentation 92.6% Bone loss detection 97%	51
Amasya et al 2023,^9^ Turkey	6000 panoramic and additional from archive 100 panoramic to compare 3 clinicians	Mask R-CNN using pre-trained ResNet-101 and Cascade RCNN	Ground truth: Three clinicians evaluated bone loss on 100 panoramic radiographs, and then model results were compared to this ground truth. Mask R-CNN is used for teeth detection, segmentation, and numbering. Cascade RCNN detected bone loss. The pretrained model tested on 100 panoramic and compared it to 3 clinicians. The model showed high accuracy in detecting and numbering teeth. It showed high accuracy detecting and staging alveolar bone loss. The model achieved binary bone loss classification metrics above 0.95.	**Teeth detection:** F-score 0.948, Accuracy 0.977, Cohen’s kappa 0.933. **Overall binary bone loss detection:** F-score 0.948, Accuracy 0.977, Precision 0.996, Recall 0.94 Cohen’s kappa 0.933 **Multiclass healthy and 3 stages bone loss:** F-score 0.996, Accuracy 0.993, Precision 0.995, Recall 0.996, Kappa 0.974 *P*-value χ2 = 0.000	48.5
Widyaningrum et al 2022,^22^ Indonesia	100 panoramic radiographs	Multi-Label U-Net and Mask R-CNN	Ground truth: A dentist and a periodontist annotated the panoramic images and stage radiographic bone loss. Both models used for segmentation and bone level detection. Multi-Label U-Net is semantic segmentation and produced superior results in image segmentation. However, bone loss detection was limited to a block of teeth rather than induvial teeth. Mask R-CNN has instance segmentation and provided superior performance in periodontitis staging and comparison with ground truth. The overall results of the models were acceptable and above 86%. The best results were seen in Stage IV alveolar bone loss with Mask R-CNN.	**1. Dice coefficient score:** U-Net = 0.97 Mask RCNN = 0.87 **2- IoU score:** U-Net = 0.98 b- Mask R-CNN = 0.74 **3- Mask-R-CNN healthy-stage IV average performance:** Precision = 0.86 Recall = 0.88 F1-Score = 0.87 **For stage IV:** Accuracy 0.95 Precision 0.85 Recall 0.88 F1-score 0.86	54
Tsoromokos et al 2022,^38^ The Netherlands	446 periapical radiographs	CNN Model not specified.	Ground truth: A dentist annotated the images, and the previous diagnosis was collected from patient files. CNN model was also compared to the dentist-annotated diagnosis. Percentages of alveolar bone loss (%ABL) detection were evaluated and compared to the dentist. Wide range of reliabilities in detecting %ABL was observed, ranging from poor in molars, moderate for premolars and good anterior teeth. The reliability was good for non-angular defect and ≥33% ABL. However, it was very poor for angular defect and poor for <33% ABL. The overall model accuracy was about 80% and it showed high sensitivity, but low specificity compared with %ABL provided by the dentist.	**CNN vs. dentists using interclass correlation (ICC):** All Teeth 0.60 *P* < .001. Non-molars 0.763 *P* < .001 Incisors 0.889 *P* < .001 Canines 0.701 *P* < .001 Premolars 0.581 *P* < .001 Molars 0.245 *P* < .048 Angular defects 0.041 Non-angular defects 0.74 <33% ABL 0.431 ≥ 33% ABL 0.641 **For <33% and ≥33% bone loss CNN performance:** Sensitivity = 0.96, Specificity = 0.41, Accuracy = 0.80	63
Shon et al 2022,^39^ Korea	4097 panoramic radiographs 2 sources: 1- Hospital 87. 2- Online AIHub 4010	U-Net and YOLOv5	Ground truth: One specialist annotated the images. U-Net detects bone level and CEJ, while YOLOv5 is used to detect teeth numbering and staging the disease. U-Net model showed high accuracy in detecting periodontal bone level (PBL) and CEJ. The model achieved variable accuracies in detecting and staging periodontitis. The integrated model showed high accuracy in staging alveolar bone loss, and periodontitis. The model achieved high sensitivity for all stages but stage 3, and high precision for all stages but stage 4. F1 score is high for stage 1 and 2 and moderate for stage 3 and 4.	**Integrated model performance metric for staging periodontitis:** Recall 0.81. Precision overall 0.73. F1-score overall 0.70. Accuracy 0.928.	52
Lee et al 2022,^40^ USA	1247 periapical radiographs	Variations of U-Net (U-Net with CNN, ResNet-34, and ResNet-50 encoder)	Ground truth: Three periodontists annotated the images and establish the diagnosis based on images and clinical records. The model was developed to segment teeth, alveolar bone, and CEJ to detect and measure radiographic bone loss (RBL) on periapical radiographs. It achieved good results in the landmarks segmentation process and high accuracy in staging radiographic bone level. It provides calculations for the distance from the CEJ to bone level. The model results were not significantly different from dental examiners but significantly faster.	**Tooth, CEJ, and bone area segmentation:** Average dice similarity coefficient above 0.91 **RBL assignment:** AUROC: 0.92 Sensitivity: 0.88 Specificity: 0.96 Accuracy: 0.94 **RBL measurement for CNN vs. 3 dental examiners** *P* > .5.	62.5
Kearney et al 2022,^41^ USA	Periapical and bitewing radiographs. a. Training 80 326 images, b. Validation 12 901 images, & c. Test and compare 10 687. T-images = 103 914	Generative adversarial networks (GANs) coupled with partial convolutions. 1. Algorithm method 1 is Deep lab V3+ 2. Algorithm method 2 is DERT.	Ground truth: Three expert clinicians annotated the images to establish the ground truth based on the clinical records. A blinded data scientist was provided with the annotated images without the clinical data. GANs were used to measure clinical attachment level (CAL) on periapical and bitewing radiographs using inpainted and non-inpainted methods. The accuracy of the methods was compared to 3 clinicians. The accuracy of GANs in detecting CAL on the radiographs was improved by inpainted method. Both mean absolute error (MAE) and Dun’s pairwise test show statistically superior results favoring inpainted over non-inpainted method in CAL prediction accuracy on bitewing radiographs.	**Mean absolute error (MAE):** Inpainted 1.04 mm and non-impainted 1.50 m **Dunn’s pairwise best value** -63.89. ***P* values** < .05	56.5
Jiang et al 2022,^42^ China	640 panoramic radiographs	U-Net and YOLO-v4 U-Net is used for automatic teeth detection and segmentation, while YOLO-v4 used for keypoint identification and alveolar ridge and furcation defect detection.	Ground truth: Three periodontists established the ground truth by annotating the images, and 3 dentists were utilized for comparison. The model showed overall acceptable accuracy superior to general dentists in staging periodontal bone loss, the highest accuracy noted in the maxillary molars and mandibular anterior teeth. The model had high specificity and accurately detected the true negative. It has acceptable accuracy in furcation bone loss detection but relatively low specificity and high accuracy in vertical absorption.	**%PBL model performance:** Accuracy 0.77, Precision 0.77, Sensitivity 0.77, Specificity 0.88, F1 0.77, & AUC 0.83 **YOLO-v4 in vertical PBL:** AP 0.52 Precision 0.88, Sensitivity 0.51, F1 0.64. **YOLO-v4 furcation PBL:** AP 0.74 Precision 0.94, Sensitivity 0.75, F1 0.64.	59
Chang et al 2022,^43^ USA	1836 periapical radiographs	Inception V3	Ground truth: Three periodontists annotated the images and established radiographic bone classification. The model achieved high performance accuracy in binary classification of periodontal bone loss, mild and severe due to limited sample size. Five-fold accuracy tests showed no statistical significance indicating valid results not circumstantial occurrence (*P* > .05).	**Accuracy of the model:** Mean accuracy all 5-folds = 0.87. *P* value > .05 **Performance parameters for 5-folds (means):** a. Sensitivity = 0.86 b. Specificity = 0.88 c. PPV = 0.88 d. NPV = 0.86 **Mean AUCROC 5-folds:** AUC = 0.92.	63.5
Alotaibi et al 2022,^30^ KSA	1724 periapical radiographs, anterior maxillary and mandibular teeth.	VGG-16	Ground truth: Three experts annotated images and established stages of radiographic bone loss. The model shows fair accuracy and binary classification of the disease as healthy or non-healthy; and multi-class classification as normal/healthy, mild, moderate, and severe. The accuracy of the model was highest for normal followed by mild, moderate, and severe. The model performance greatly varies with multi-class correlation, with significant differences noted between all parameters. Moderate agreement between ML and periodontists for binary classification as shown by Cohen Kappa of 0.51 and fair agreement for multiclass correlation k = 0.41. The model best results showed its ability to differentiate bone loss from no bone loss cases. The model showed acceptable sensitivity and specificity values with slightly superior specificity for binary classification of alveolar bone.	**Model Accuracy:** Binary 0.73 Multi-class 0.59 Binary vs. multi-class classification *P* < .05. **Model performance Binary classification,** **Normal vs. abnormal:** Weighted average for all binary: Precision, recall, and F1-scores is 0.73. **Multi-class classification, Mild, Moderate, Severe:** Weighted average: Precision 0.60, Recall 0.59, F1 0.59. **Healthy vs. Diseased alveolar bone:** Sensitivity 0.73 Specificity 0.79	55.5
Li et al 2021,^44^ China	506 panoramic radiographs; 2 hospitals, Suzhou and Zhongshan.	Deetal-Perio	Ground truth: Radiographs used were previously diagnosed by dentists. One dentist labelled and annotated the images. Deetal-Peri achieved promising results in segmentation and numbering methods with mean average precision (mAP) and dice coefficient values above 80%. Both segmentation and disease prediction tasks achieved better results outperformed previously published models with high accuracy >80% on data from 2 hospitals. The authors stated high performance in periodontitis stages, but the provided results are for overall values and no specific stages provided.	**Dental segmentation and numbering:** a. Suzhou: mAP 0.863, Dice (all) 0.892, and Dice (single) 0.809. b. Zhongshan: mAP 0.927, Dice (all) 0.903, and Dice (single) 0.819. **Performance for periodontitis:** a. Suzhou: Macro F1 0.889, Accuracy 0.892 b. Zhongshan: F1-score 0.812, Accuracy 0.819	53.5
Kabir et al 2021,^20^ USA	700 periapical radiographs, + (10 cases * 10-12 PA) used for additional testing.	HYNETS (Hybrid NETwork for pEriodoNTiTiS STagES from radiograpH).	Ground truth: Three examiners annotated the images and assigned the stages of radiographic bone level. The model integrates segmentation and periodontitis classification tasks. It achieved high accuracy for teeth and alveolar bone segmentation and periodontitis staging. The model outperformed multiple published models.^45–48^ Compared to clinicians, it showed highest agreement with periodontal professor followed by periodontology resident and clinical periodontist. No significant difference (*P* = .42) between the RBL percentage measured by experts and HYNETS.	**Dice similarity measured coefficient (DSC) for segmentation:** Bone area: DSC 0.96 Teeth: DSC 0.95, and CEJ: DSC 0.91, **Accuracy measured with AUC-ROC:** Stage I: AUC = 0.99, Stage II: AUC = 0.93, Stage III: AUC = 0.96. **Cohen’s Kappa between the model and 3 periodontists:** HYNETS vs. professor κ = 0.6998 HYNETS vs clinical periodontist k = 0.4712 HYNETS vs period-student k = 0.4959 **Model vs. clinicians’ periodontitis staging** *P* > .05.	54.5
Danks et al 2021,^49^ UK	340 periapical radiographs	Symmetric hourglass architecture with ISM model	Ground truth: landmarks annotation and bounding box were drawn by 2 periodontal residents. The model is used to detect radiographic bone loss (RBL) through landmark localization (CEJ, apex, and bone level). Landmarks were best localized for single-rooted teeth. The best-localized landmark was the CEJ and the worst was the apex. It achieved high accuracy for landmark localization with about 58% severity staging, which aligns with clinicians.	**Percentage correct keypoints (PCK) for landmark localization:** Single root 88.9% Double roots 73.9% Triple roots 74.9% PCK all root 83.3%. **Average PBL accuracy evaluation of the model compared to clinicians’ visual evaluation for full radiographs:** Average PBL error 10.69%. Severity stage accuracy calcification accuracy 58%.	62
Chen et al 2021,^50^ China	2900 periapical radiographs	Faster R-CNN′	Ground truth: An experienced dentist drew the bounding box around each disease area. Several dental pathologies evaluated that are caries, apical periodontitis, and periodontal periodontitis. This study focused on reporting periodontal periodontitis. The model achieved good accuracy close to the ground truth with limited misdiagnosis. The model was able to detect all stages (mild, moderate, and severe), with severe periodontitis cases detection that is better than severe caries and severe apical periodontitis. Training strategy, disease category, and disease severity significantly affected the mode performance (*P* < .001).	**Baseline for periodontal periodontitis:** IoU 0.68, Precision 0.56, Recall 0.62, AP 0.44. **For Net-A:** IoU 0.68, Precision 0.57, Recall 0.61, AP 0.45. **For net B&C, stages:** **Mild:** IoU 0.68, Precision 0.49, Recall 0.55, AP 0.39. **Moderate:** IoU 0.70, Precision 0.42, Recall 0.47, AP 0.27. **Severe:** IoU 0.70, Precision 0.47, Recall 0.49, AP 0.35.	47.5
Thanathornwong et al 2020,^51^ Thailand	100 panoramic radiographs	Faster R-CNN (base CNN was ResNet-101).	Ground truth: Periodontally compromised teeth annotated by 3 experts The model was used to detect periodontally compromised teeth (PCT) with high accuracy >80%. Only moderate and severe cases were used due to the limited sample (Binary classification). There is a significant overlap between the boxes detected by the CNN and the ground truth.	**Ground truth detection of PCT vs. no-detection of healthy:** average precision rate (APR) = 0.81 and average recall rate (ARR) = 0.80 **Detection of PCT vs. healthy:** Sensitivity of 0.84, Specificity of 0.88, and F-measure of 0.81.	50
Moran et al 2020,^27^ Brazil	467 periapical radiographs	ResNet 50 and inception. A third SVM used for comparison.	Ground truth: Two expert dentists annotated the images and established healthy and bone loss cases. The models were used to detect periodontal bone loss from healthy teeth on PA radiographs. Both models demonstrated high performance in detecting periodontal bone loss. Inception models showed superior accuracy results compared to ResNet 50 and SVM. Incorrect identification was mostly type I error, false positive.	**ResNet:** Precision 0.74, Sensitivity 0.75, Specificity 0.73, NPV 0.745, AUC-ROC 0.864, AUC-PR 0.868. **Inception:** Precision 0.76, Sensitivity 0.92, Specificity 0.71, NPV 0.90, AUC-ROC 0.86, AUC-PR 0.85 **SVM:** Precision 0.54, Sensitivity 0.85, Specificity 0.24, NPV 0.64, AUC-ROC 0.51, AUC-PR 0.55.	54.5
Kurt et al 2020,^52^ Turkey	2276 panoramic radiographs.	Pretrained Google Net Inception v3	Ground truth: Two specialists annotated the images and determined bone loss on the images. The model achieved high performance in detecting alveolar bone loss and health cases. All performance parameters achieved results above 88%. The model showed high sensitivity and accuracy results.	**Performance:** Sensitivity 0.94, Specificity 0.86, Precision 0.89, Accuracy 0.91, F1-Score 0.92.	49.5
Chang et al 2020,^45^ South Korea	340 panoramic radiographs.	Modified CNN from Mask R-CNN based on a feature pyramid network (FPN) and a ResNet101 backbone	Ground truth: The area enclosed bone level, teeth, and CEJ were delineated by oral and maxillofacial radiologists, numbers weren’t specified. Additionally, the comparison between the model and 3 clinicians was performed. The model used 2 stages method, detect landmarks (Teeth, Bone, and CEJ) and calculate percentage of alveolar bone loss (%ABL). The accuracy and reliability of the model was comparable to clinicians, with no significant difference between bone loss measured by CNN and 3 dentists (professor, fellow, resident). The accuracy of incisors and molars was lower than canines and premolars. Pearson’s (PCC) and interclass (ICC) correlation coefficients show a strong correlation between the model and clinicians’, highest results were between the model and the professor.	**Landmarks detection: Pixel Accuracy (PA), dice coefficient (DC), and Jaccard index (JI):** Bone level: JI 0.92, PA 0.93, DC 0.88. CEJ level: JI 0.87, PA 0.91, DC 0.84. Teeth: JI 0.87, PA 0.91, DC 0.83. **Mean absolute difference (MAD) of periodontitis stages, whole jaw:** Prof 0.21, Fellow 0.25, Resident 0.25, CNN 0.25, Overall MAD 0.25 *P* > .05 **Pearson’s correlations (PCC) CNN vs.:** Professor 0.76, Fellow 0.73, Resident 0.70 Overall PCC 0.73. whole jaw (*P* < .01). **Interclass correlations (ICC) CNN vs.:** Professor 0.86, Fellow 0.84, Resident 0.82 Overall PCC 0.91. Whole jaw (*P* < .01).	54.5
Krois et al 2019,^47^ Germany	85 panoramic radiographs	Custom made CNN	Ground truth: Three examiners determined the points landmark for alveolar bone loss measurements The models was also compared to 6 dentist of different specialties. However, PCC or ICC weren’t provided. The CNN model showed comparable results to 6 dentists. Different cut-offs values (20%, 25%, and 30%) of periodontal bone loss were used. These cut-offs significantly affected the performance parameters. Drastic decrease in the specificity of the dentists with only slight increase in the sensitivity and NPV at higher cut-offs values. Higher cut-offs have a limited effect on the model’s accuracy. The model sensitivity is reduced at higher cut-offs compared to dentists.	**Model performance at 20% cut-off of PBL (mean values):** Accuracy 0.81, Sensitivity 0.81 Specificity 0.81 F1- score 0.78 Precision 0.76 NPV 0.85 **Performance of 6 dentists:** Accuracy 0.76, Sensitivity 0.92 Specificity 0.63 Fleiss kappa 0.52 (moderate between 6 dentists). **ROC also calculated for all the examiners and CNN:** Average ROC curve AUC 0.89.	62
Kim et al 2019,^46^ South Korea	12 179 panoramic radiographs 800 panoramic used for testing and comparisons.	DCNN called DeNTNet	Ground truth: Five dental hygienists monitored by a maxillofacial surgeon annotated the images. The study evaluated the periodontal bone loss as binary (present or absent) and compared 5 experienced dental hygienists. It uses muti-step to detect bone level and teeth numbering. The model achieved high accuracy compared to experienced clinicians at baseline without segmentation and transfer learning. However, the model F1-score outperformed the clinicians when regional segmentations and transfer learning were used. The model performance for third molars was lower compared to dental clinicians. Different testing modes were used in the study. Multiple DeNTNet modes were evaluated such as high sensitivity setting and high spasticity setting to increase sensitivity or specificity, but this results in reduced other parameters.	**The average performance of 5 dental clinicians:** AUROC 0.85, F1-score 0.69, sensitivity 0.78, specificity 0.92, PPV 0.62, NPV 0.96. **DeNTNet (Baseline)** AUROC 0.85, F1-score 0.69, sensitivity 0.78, specificity 0.92, PPV 0.62, NPV 0.94. **DeNTNet (Balanced setting):** AUROC 0.95, F1-score 0.75, sensitivity 0.77, specificity 0.95, PPV 0.73, NPV 0.96.	66
Lee et al 2018,^48^ Korea	1740 periapical radiographs	Modified VGG-19 network architecture.	Ground truth: Three periodontists categorized the images. The study evaluated the diagnosis of periodontally compromised teeth (PCT) and predicted hopeless teeth. The model was compared to 3 board-certified periodontists. The model showed high diagnostic accuracy for PCT, and highest values for severe cases. CNN tends to judge PCT as severe. The prediction AUC was higher for CNN in the premolar area and higher for periodontists in molar areas. This may be due to complicated structures in the molar region. However, this was not significant. The CNN model had comparable results to board-certified periodontists.	**1. CNN diagnosis accuracy of PCT** **For premolars:** Overall accuracy 0.81 Moderate PCT 0.773 Severe PCT 0.828 **For molars:** Overall accuracy 0.767 Moderate PCT 0.703 Severe PCT 0.813 **2- Prediction accuracy for hopeless teeth by CNN and periodontists** **For premolars:** CNN accuracy 0.828 CNN AUC 0.826 Periodontists’ accuracy 0.797 Periodontists’ AUC 0.793 **For molars:** CNN accuracy 0.734 CNN AUC 0.73 Periodontists’ accuracy 0.797 Periodontists’ AUC 0.793	61

### Results of individual studies

The majority of the studies used panoramic radiographs to assess radiographic bone and periodontal disease through a DL approach.^3^^,^^4^^,^^9^^,^^22^^,^^31^^,^^32^^,^^34^^,^^35^^,^^39^^,^^42^^,^^44–47^^,^^51^^,^^52^ Periapical radiographs were used in 13 papers.^20^^,^^26^^,^^27^^,^^30^^,^^33^^,^^36–38^^,^^40^^,^^41^^,^^43^^,^^48–50^ Only one article used bitewing radiographs.^41^

Twenty-nine studies used AI models to assess radiographic bone loss on dental radiographs.^3^^,^^4^^,^^9^^,^^20^^,^^22^^,^^26^^,^^27^^,^^30–40^^,^^42–52^ Kearney et al^41^ used GANs to evaluate inpainted and non-inpainted methods, which were used to evaluate clinical attachment loss rather than radiographic bone loss. Although this study did not assess the bone level, it still aligns with the original inclusion criteria and keywords established at the start of the search, which were never modified after the search process. It evaluated periodontal disease/periodontitis on 2D dental radiographs using an AI model. Therefore, it was not excluded from the review.

As seen in Table 1, all studies included in this review have established ground truth through expert image annotations and labeling. However, only 14 studies compared model performance to clinical experts.^9^^,^^20^^,^^26^^,^^32^^,^^34^^,^^36^^,^^38^^,^^40–43^^,^^46–48^

### Risk of bias assessment

Based on the quantitative analysis of the APPRAISE-AI tools,^25^ 7 papers were considered high quality (scored 60-79): Liu et al 2023; Tsoromokos et al 2022; Chang et al 2022; Lee et al 2022; Danks et al 2021; Kim et al 2019; and Krois et al 2019.^34^^,^^38^^,^^40^^,^^43^^,^^46^^,^^47^^,^^49^ Nineteen papers were considered intermediate quality (score 50-59), and 4 papers scored below 50 and were considered low-quality papers. The lowest scoring paper (Sameer et al 2023^31^) scored 35, which is considered very low quality. Table 2 summarizes the papers based on the AI-score rating. The mean score of all items was 55.3 (median = 54.5; SD = 7.2).

**Table 2.** Number and percentage of articles in each quality category via overall scores from the APPRAISE-AI critical appraisal tool.^25^

Quality category	Number (%)	Papers
High quality (60 ≤ Score < 80)	7 (23.3%)	Liu (2023), Tsoromokos (2022), Chang (2022), Lee (2022), Danks (2021), Kim (2019), Krois (2019).
Intermediate quality (50 ≤ Score < 60)	19 (63.3%)	Kong (2023), Karacaoglu (2023), Saylan (2023), Vollmer (2023), Ryu (2023), Chen (2023)—first, Chen (2023)—second, Alotaibi (2022), Li (2021), Kabir- (2021), Moran (2020), Chang (2020), Widyaningrum (2022), Shon (2022), Lee (2018), Kearney (2022), Jiang (2022), Kurt (2020), Thanathornwong (2020)
Low quality (40 ≤ Score < 50)	3 (10.0%)	Mao (2023), Amaysa (2023), Chen (2021)
Very low quality (Score <40)	1 (3.3%)	Sameer et al 2023

As noted above, the APPRAISE-AI tool splits the appraisal of each paper into distinct sections, namely, title/introduction, methods, results, conclusions, and others. Each APPRAISE-AI item was mapped to one of the following domains: clinical relevance, data quality, methodological conduct, robustness of results, reporting quality, and reproducibility. In order to compare results for domains against each other using the same scale, note again that we scale domain scores linearly to lie in the range [0,100], where: clinical relevance, mean = 97.1 and SD = 6.3; data quality, mean = 58.9 and SD = 9.4; methodological conduct, mean = 54.4 and SD = 11.6: robustness of results, mean = 42.7 and SD = 10.1; reporting quality, mean = 72.9 and SD = 16.1: and reproducibility, mean = 45.5 and SD = 11.4. Table 3 also summarizes the results of each domain. As seen, most papers are either intermediate or low-quality (ie, domain score < 60), and only 7 papers produced high-quality results (ie, 60 ≤ domain score < 80).

**Table 3.** Domain scores scaled in the range 0 (extremely poor) to 100 (extremely good) using the APPRAISE-AI critical appraisal tool.^25^

Scaled	All items	Clinical relevance	Data quality	Methodological conduct	Robustness of results	Reporting quality	Reproducibility
Mean	55.3	97.1	58.9	54.4	42.7	72.9	45.5
Median	54.5	100.0	58.3	56.3	45.0	75.0	47.5
SD	7.2	6.3	9.4	11.6	10.1	16.1	11.4

### Results of syntheses

**Figure 2.**
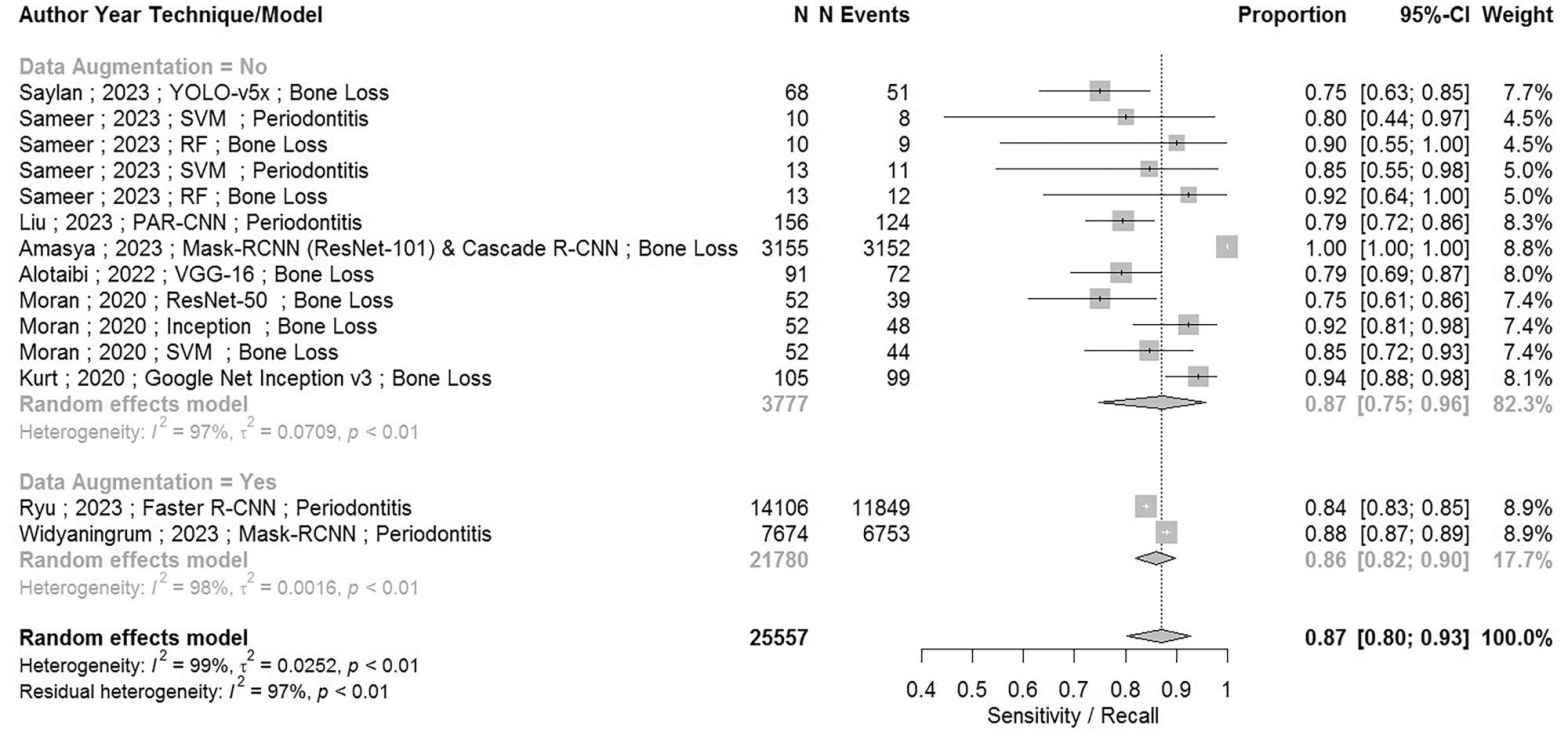
Forest plot including meta-analysis for the model performance measure: sensitivity.

**Figure 3.**
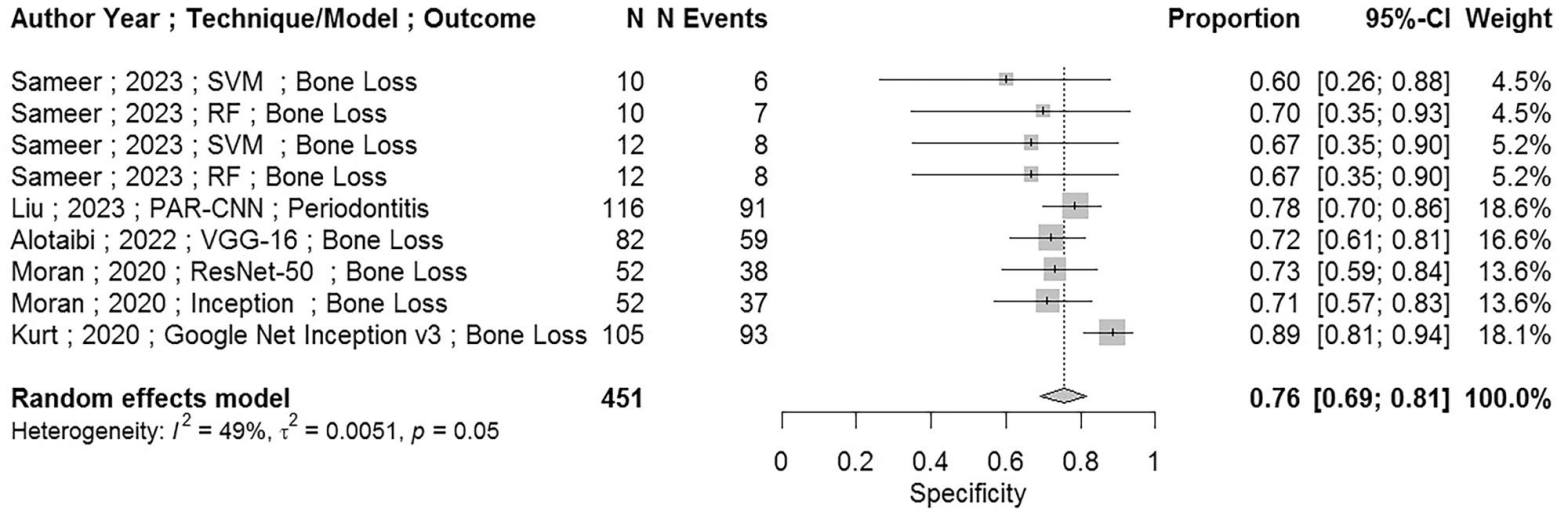
Forest plot (excluding Moran [2020]) with meta-analysis for the model performance measure: specificity.

Eleven papers^4^^,^^9^^,^^22^^,^^27^^,^^30–34^^,^^40^^,^^52^ were found to be eligible for the meta-analysis. However, Mao’s et al 2023^33^ study was removed from the meta-analysis, because it used a training sample for testing model performance instead of a validation sample, indicating a high risk of bias. Therefore, 10 papers^4^^,^^9^^,^^22^^,^^27^^,^^30–32^^,^^34^^,^^40^^,^^52^ have been used in the analysis. There was enough data for 6 measures to be assessed through meta-analysis. Table 3 shows results for the (point) estimates (and 95% CI) for sensitivity (recall), specificity, accuracy, precision (PPV), NPV, and F1-score. Figures 2 and 3 show forest plots for sensitivity and specificity, respectively. Additional figures of forest plots are attached as Supplementary Material. All results show overall high values for all parameters (ie, sensitivity (recall), specificity, accuracy, PPV, NPV) and F1-score.

Results of meta-analysis for the sensitivity are shown in Figure 2 and Table 4, where sensitivity is given by 0.87 (95% CI, 0.75-0.96) for non-augmentation cases, 0.86 (95% CI, 0.82-0.90) for augmentation cases, and 0.87 (95% CI, 0.80-0.93) for both augmented and non-augmented cases combined. Note that we consider the data to be augmented when both training and testing data used in the model are augmented. Although confidence intervals are much narrower for the augmented data (as expected given that sample sizes are much larger), no compelling differences were identified in the point estimates between augmented and non-augmented data. There were inconclusive differences by outcome type and no difference by type of data measured for testing and training. As demonstrated, our approach acknowledges that finite test sample size itself impacts the confidence intervals in the meta-analysis. We demonstrated how augmentation reduced the confidence intervals by conducting a subgroup analysis on augmented versus non-augmented data.

**Table 4.** Overview of the meta-analysis results.

Measure	Description of measure	**Point estimate and 95% CIs from meta-analysis** (expressed as percentages here)
Sensitivity (also known as Recall)	Percentage of cases with periodontitis that were classified correctly as positive	87% (95% CI, 80%-93%)
Specificity	Percentage of cases without periodontitis that were classified correctly as negative	76% (95% CI, 69%-81%)
Accuracy	Percentage of cases both with and without periodontitis that were classified correctly	84% (95% CI, 75%-91%)
PPV (also known as Precision)	Percentage of positive classifications (periodontitis etc.) that were correct	81% (95% CI, 77%-84%)
NPV	Percentage of negative classifications (no periodontitis etc.) that were correct	81% (95% CI, 73%-88%)
F1-score	Harmonic mean of the precision and sensitivity	80% (95% CI, 74%-85%)

Results of meta-analysis for the specificity are shown in Figure 3 and Table 4. None of the studies used in meta-analysis for the specificity employed data augmentation. Results of meta-analysis for the specificity were 0.69 (95% CI, 0.56-0.80). Moran *et al*’s (SVM)^27^ study was identified as an outlier and a sensitivity analysis was carried out. Removing this study reduced heterogeneity and changed the results of meta-analysis for the specificity slightly to 0.76 (95% CI, 0.69-0.81) and results are shown in Figure 3.

Meta-analysis was carried out for accuracy, PPV, NPV, and F1 score without data augmentation. Results for the accuracy were 0.82 (95% CI, 0.72-0.90) without augmentation across all studies. Removing the potential outlier of Moran *et al*’s (SVM; which did not use data augmentation),^27^ the accuracy changed slightly to 0.84 (95% CI, 0.75-0.91) across all studies, thus indicating the minimal impact of this potential outlier. Results for the precision (PPV) were 0.75 (95% CI, 0.67-0.83) without augmentation across all studies. Removing the potential outlier of Moran *et al*’s 2020 (SVM; which did not use data augmentation)^27^ reduced heterogeneity between studies and enabled the use of fixed-effects meta-analysis, PPV was adjusted to 0.81 (95% CI, 0.77-0.84) across all studies. Results for the NPV were 0.81 (95% CI, 0.73-0.88) across all studies. Results for the F1-score were 0.80 (95% CI, 0.74-0.85) across all studies. Again, note that results for all of these performance measures and across all studies are shown in Table 4 and Supplementary Materials.

## Discussion

A systematic review was carried out here of the application of DL to detect periodontitis and periodontal bone loss from radiographic dental images. The review adhered to PRISMA standards, where 30 papers were used in this review. All articles were critically appraised using the APPRAISE-AI by 2 independent reviewers (ie, 2 authors of this article). Measures of model performance are often ratios of 2 integers, where this ratio lies in the range 0 to 1 and had a meaningful interpretation, namely: a value near to zero indicating extremely poor performance and near to 1 indicating extremely good performance. Standard methods of meta-analysis for a proportion using the “metaprop” command in R V3.6.1 could therefore be employed. Eleven papers provided quantitative evidence amenable to meta-analyses, and results were presented for the sensitivity (aka recall), specificity, accuracy, positive predictive value (aka precision), NPV, and F1 scores.

Using boundaries set by the APPRAISE-AI tool,^25^ critical appraisals indicated that 1 out of 30 papers (3.3%) were of very low quality (score < 40), 3 (10.0%) were of low quality (40 ≤ score < 50), 19 (63.3%) were of intermediate quality (50 ≤ score < 60), and 7 (23.3%) were of high quality (60 ≤ score < 80). No papers were of very high quality (score ≥ 80). This shows broadly that the quality of papers was adequate on the whole, although there was some variation in quality. The APPRAISE-AI tool subdivided the papers into 5 key areas/domains, namely, clinical relevance, data quality, methodological conduct, robustness of results, reporting, and reproducibility.

Not surprisingly, virtually all papers scored well on clinical relevance. This is probably because the maximum domain score was only 4 and so any attempt at the title, background, objective and problem, and clinical implementation was likely to receive a mark each. Previous systematic reviews show similar results as authors tend to have good reporting of clinical relevance and implementation and provide clear background and objectives.^53^^,^^54^ Similarly, reporting quality had a fairly high score compared to the other domains, and again its maximum score was only 12. Also, reporting cohort characteristics, limitations, and disclosures ought to be fairly straightforward tasks, and some form of “critical analysis” is a very common task when writing a paper, as noted in other reviews.^24^^,^^54^^,^^55^

Methodological conduct and data quality ought also to be straightforward tasks, but scoring for these domains was slightly lower. It is noticeable that many papers scored poorly on stating the sources of their data and also slightly less well on eligibility criteria for the data quality domain. Not surprisingly, authors tended to explain what the ground truths were and how data were abstracted and prepared, which are both “bread and butter” tasks in image analysis using DL. Similarly, data splitting and sample size calculations were explained adequately for the methodological conduct domain, although baseline models were explained less well. It seems that other reviews identified similar issues in explaining the methodological conduct with increased risk of bias despite using different critical appraisal tools.^23^^,^^24^^,^^55^

In addition, more than 50% of the included papers, in which experts annotated the images, did not include direct and blinded clinicians’ comparison.^3^^,^^4^^,^^22^^,^^27^^,^^30^^,^^31^^,^^33^^,^^35^^,^^37^^,^^39^^,^^44^^,^^45^^,^^49–52^ This limits the ability to validate the models’ performance in real-world experience. It might be argued that image annotation by experts can serve as a baseline to which an AI model is compared and validated. However, it is crucial to compare AI models’ performance directly with that of blinded experts, especially trained oral and maxillofacial radiologists, to ensure that they can complement and enhance clinicians’ diagnostic accuracy and improve trust and reliability.

Finally, robustness and reproducibility domains scored badly. For robustness, all items in this domain scored somewhat poorly, although error analysis was particularly poor where only a few papers even considered this. This reflects previous findings that highlight a lack of transparency and thoroughness in these areas.^23^^,^^24^^,^^33^^,^^54^^,^^55^ The poorer score for the reproducibility domain was driven by a lack of transparency (eg, authors not providing links to code or data) because model description and model specification tended to be reported extremely well. Overall, our analysis produced similar results to the APPRAISE-AI tool^25^ that showed the lowest domain scores were robustness of results, reproducibility, and methodological conduct.

Results of measures of model performance (sensitivity, specificity, F1 etc.) showed that overall performance was quite good, although there is quite a lot of variation between studies and so there is some “room for improvement,” which is consistent with previous studies.^23^^,^^24^^,^^55^ There was some evidence of a difference between sensitivity and specificity. Notably, results for the specificity of 76% (95% CI, 69%-81%) were somewhat lower than results for the sensitivity of 87% (95% CI, 80%-93%), indicating broadly that classifying negative cases correctly (ie, without disease) was a harder task than classifying positive cases correctly (ie, with disease). 95% confidence intervals were much smaller for augmented data compared to non-augmented data, which is exactly what one would expect as sample sizes have been increased synthetically compared to non-augmented data. Point estimates for these measures were broadly about the same (or perhaps slightly higher in some cases) for augmented versus non-augmented data, although this was inconclusive here. There was no evidence from this analysis that a particular type of neural network/DL model performs better than the others, although this might emerge in the future. Indeed, there appeared to be no other strong factor affecting results for measures of model performance, as far as we could tell.

One strength of the analyses carried out here is that we carry out meta-analysis for measures of model performance for AI applied to dental images. Furthermore, we have used an explicit critical appraisal tool to analyse our sources, which is another advantage. Weaknesses of our analyses are that there were relatively few studies for meta-analyses, although this is fast-moving field. Finally, we found high heterogeneity in our data, which makes the results of meta-analysis less reliable, even despite using random effects meta-analyses and sensitivity analyses. A common criticism of meta-analysis for experimental or lab-based studies is that the diverse setups (methods, populations, outcomes, etc.) render any average or composite value meaningless. However, our perspective is that meta-analysis remains valuable for gaining an overall understanding of results, as the data patterns for these measures generally show consistency across different studies.

In relation to clinical practice, rapid progress is clearly being made in this field. The results of meta-analyses for all of the measures of model performance indicate that (on average) models are not good enough as an automated screening tool as yet. Common acceptable performance cut-off values for screening tests are often cited as sensitivity and specificity roughly greater than or equal to 80%-90%,^56^ although one should note that the precise levels for these cut-offs are also strongly case-dependent and/or disease-dependent.^56–58^ However, we remark that some of the models in the papers used in this study might indeed perform well according to these criteria, but probably also require more (external) validation and testing. Critical appraisal carried out here indicates that a lack of transparency, absence of analysis of outliers and errors, and opacity regarding data sources in the articles considered here are potentially significant barriers to the subsequent adoption and translation by the dental community.

Future research should focus on transparency and rigorous explanation of study design and methods used in performing AI studies. We believe that the newly developed APPRSISE-AI tool by Kwong et al^25^ provides a useful tool to analyse the quality of future AI studies, including potential risks of bias. It can be used as a guideline in future research to create coherent, valid, and reproducible papers.

## Conclusion

Studies showed various DL models can be developed and applied in dento-alveolar detection and segmentation and subsequent periodontal bone level evaluation with high accuracy. We applied the new APPRAISE-AI Tool in our study as it takes into consideration all necessary information that has to be reported in AI studies. Meta-analysis results indicate that model efficacy, averaged across included studies, is generally good to very good. Data augmentation appeared to enhance model performance, but this was not statistically significant. Despite literature heterogeneity and various performance parameters, AI models evaluated alveolar bone loss on 2D dental radiographs with high efficacy. However, it may not be good enough as an automated screening tool as yet; due to the lack of transparency, absence of analysis of outliers and errors, opacity, and discrepancy within studies. Finally, this systematic review highlights the need for more rigorous standards and clear guidelines in conducting, documenting, and reporting AI research in dentistry.

## Supplementary Material

twae070_Supplementary_Data
