# Supplementary material for: Detection of periodontal bone loss and periodontitis from 2D dental radiographs via machine learning and deep learning: systematic review employing APPRAISE-AI and meta-analysis
Source: Dentomaxillofac Radiol. 2024 Dec 5;54(2):89–108. doi: 10.1093/dmfr/twae070 (PMC11979759; doi:10.1093/dmfr/twae070)
Supplement: twae070_Supplementary_Data [file twae070_supplementary_data.zip › APPRAISE-AI items and domains -- sup.docx]

**APPRAISE-AI Domains and Items**

**Clinical relevance (Domain Score = 4)**

Item 1. Title – Identify the report as an AI application to a specific clinical question. (Item score = 1)

Item 2. Background – Describe the clinical problem and rationale for developing AI models. Review existing relevant literature exploring AI models for the problem being addressed. (Item score = 1)

Item 3. Objective and problem – Clearly state what the proposed AI model(s) aims to address with respect to study population and outcome. (Item score = 1)

Item 21. Implementation into clinical practice – Describe how the AI model(s) can be applied to clinical practice, with respect to the potential to improve patient care, clinical decision-making, and/or efficiency. (Item score = 1)

**Data quality (Domain score = 24)**

Item 4. Source of data – Describe how the dataset was obtained (e.g., single/multi-center, local/national database, etc.), and study period. If relevant, the diversity of the dataset is also described (e.g., inclusion of community hospitals, low/middle income populations, and institutions from other countries). (Item score = 8)

Item 5. Eligibility criteria – Specify all criteria for inclusion/exclusion of patients and features. Provide appropriate details (e.g., adults, age > 18) and rationale. (Item score = 3)

Item 6. Ground truth – Define the ground truth of interest. Describe how it was collected (e.g., manual annotation by experts) and encoded (e.g., binary, categorical, dichotomized continuous, continuous variable, etc.). For unsupervised learning, describe what measure(s) and associated data will be used to assess cluster validity (e.g., correlating disease-specific features with overall survival). (Item score = 6)

Item 7. Data abstraction, cleaning, preparation – Describe the methods used to develop the final dataset, with consideration of the following: Feature abstraction; Handling of missing data (e.g., removal, imputation); Feature engineering; Removal of features. (Item score = 7)

**Methodological conduct (Domain score = 20)**

Item 8. Data splitting – Specify how the data was divided into the training and testing cohorts. (Item score = 7)

Item 9. Sample size calculation – Provide rationale for sample size required for model development (e.g., based on power calculation). (Item score = 5)

Item 10. Baseline – Describe the baseline model that will serve as a comparison for the AI model(s). (Item score = 8)

**Robustness of results (Domain score = 20)**

Item 15. Model evaluation – List the evaluation metrics used to assess performance and calibration, including the justification for selection. (Item score = 5)

Item 16. Clinical utility assessment – Describe appropriate metrics for readers to understand the risk/benefit trade-offs of using the AI model at the specified decision threshold (e.g., decision curve analysis) (Item score = 5)

Item 17. Bias assessment – Compare evaluation metrics for the AI model(s) and reference standard when stratified by patient- and task-specific subgroups to identify subgroups that benefit, are not helped at all, or harmed by the models. Patient-specific subgroups may include age group, gender, ethnicity, or socioeconomic status. Task-specific subgroups are disease-specific and may include risk stratification (e.g., low-, intermediate-, and high-risk disease in prostate cancer), or subtyping (e.g., different bacteria in positive blood cultures). (Item score = 6)

Item 18. Error analysis – Analyze predictive errors to identify characteristics that are more prone to inaccurate predictions. Determine if there are any surprise errors (e.g., clearly inaccurate predictions based on clinical judgement). (Item score = 4)

Item 19. Model explanation (optional) – Describe methods used to explain AI models. (Item score = 0)

**Reporting quality (Domain score = 12)**

Item 13. Cohort characteristics – Provide the total cohort size and summary statistics of the training, validation (if used), and testing cohorts, including incidence of the ground truth of interest. (Item score = 4)

Item 20. Critical analysis – Describe the main findings of the study, including: New predictors of the ground truth of interest identified using AI; Strengths of the AI model(s) compared to the current models in the literature; Why the AI model(s) performed better/worse than what is currently available; (Optional) If feature importance rankings were used, describe whether they were aligned with clinical intuition and known prognostic factors. (Item score = 5)

Item 22. Limitations – Describe how the AI model(s) can be applied to clinical practice, with respect to the potential to improve patient care, clinical decision-making, and/or efficiency. (Item score = 2)

Item 23. Disclosures – Disclose all financial relationships, sources of funding, and potential conflicts of interest. (Item score = 1)

**Reproducibility (Domain score = 20)**

Item 11. Model and processing description – Describe the AI model(s) and software libraries investigated. (Item score = 2)

Item 12. Hyperparameter tuning – Specify all model hyperparameters that were optimized, the search space for hyperparameter tuning, and evaluation metric(s) used to optimize parameters. (Item score = 5)

Item 14. Model specification – Present the final AI model and specify the final panel of features included and hyperparameters tuned. (Item score = 3)

Item 24. Transparency – Share the data, source code, or release an application that runs the code. Data dictionary involves providing descriptions of all features and ground truth, with consideration of the following: Data type (i.e., categorical or numerical); Method of collection or measurement (e.g., serum hemoglobin in g/dL); Range of values (e.g., yes or no). (Item score = 10)

**Overall APPRAISE-AI score = 100**
