# Supplementary material for: Detection of periodontal bone loss and periodontitis from 2D dental radiographs via machine learning and deep learning: systematic review employing APPRAISE-AI and meta-analysis
Source: Dentomaxillofac Radiol. 2024 Dec 5;54(2):89–108. doi: 10.1093/dmfr/twae070 (PMC11979759; doi:10.1093/dmfr/twae070)
Supplement: twae070_Supplementary_Data [file twae070_supplementary_data.zip › Meta analysis figures for online --supMao_removed.docx]

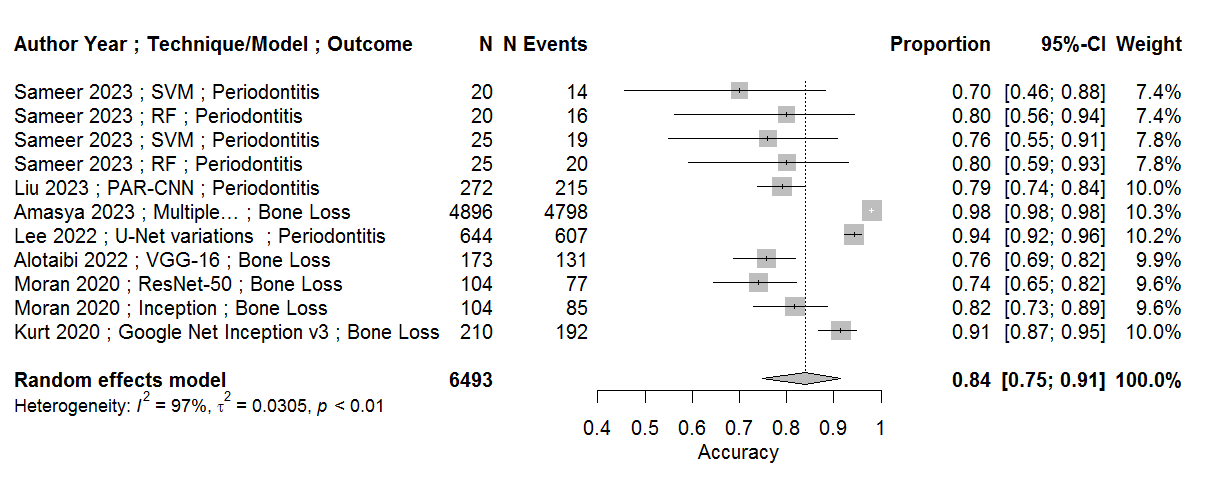


Figure: Forest plot (excluding Moran (2020) and Mao (2023)) with meta analysis for the model performance measure: accuracy


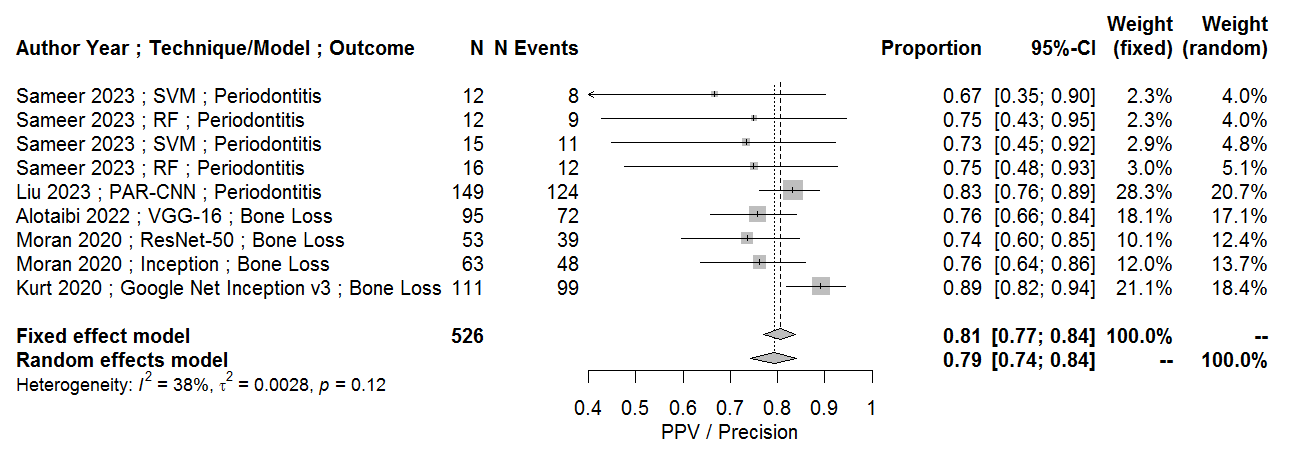


Figure: Forest plot (excluding Moran (2020) and Mao (2023)) with meta analysis for the model performance measure: Positive Predictive Value (aka Precision)


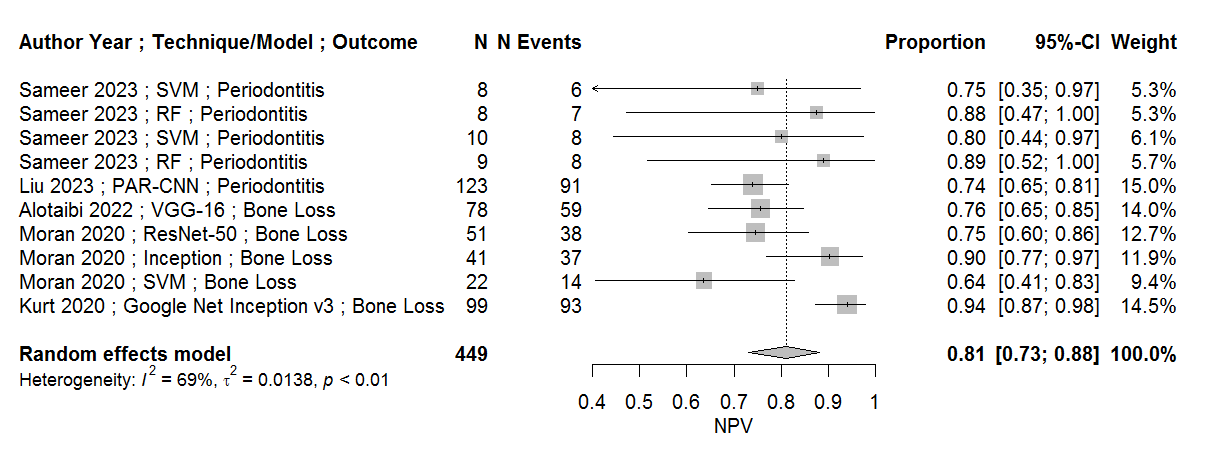


Figure: Forest plot (excluding Mao (2023)) with meta analysis for the model performance measure: Negative Predictive Value


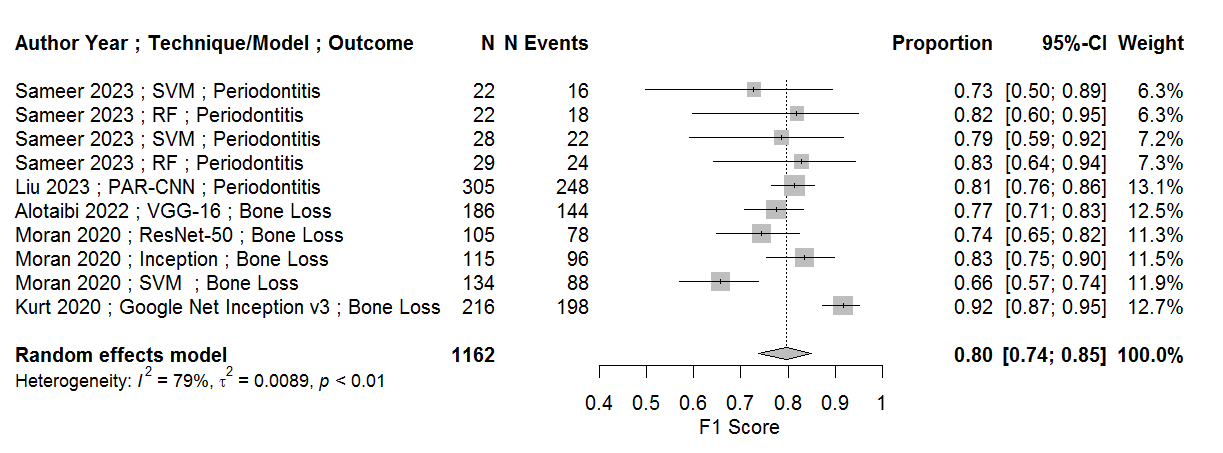


Figure: Forest plot (excluding Mao (2023)) with meta analysis for the model performance measure: F1 Score

**Overall conclusions:**

***Validity of the method:***

- We have proven that (mathematically, at the very least) meta analysis can be used to find averages of measures of model efficacy by treating the outcome measures as simple proportion / ratios.
- 95% CIs are useful to obtain some idea of how accurate these average is, although much caution must be employed as heterogeneity is often a little high.
- Note that a common criticism of meta analysis for experimental / lab-based studies, as I understand it, is that their set-ups are so different (methods, populations, outcomes etc.) than any average / composite value is meaningless. However, my feeling here is that it results of meta analysis are still useful to obtain an overview of results as the patterns in the data for these measures appear broadly consistent across the different studies.

***What do the results indicate (very broadly):***

- Values for all measures indicate (averaged over all studies) good to very good results for ALL measures of model efficacy (i.e., from 76% to 88% averaged over all studies and across the different measures).
- Notably, results for the specificity (meta analysis result = 76% (95% CI: 69% to 81%) had a lower value than sensitivity (meta analysis result = 87% (95% CI: 80% to 93%), indicating broadly that classifying negative cases (i.e., without periodontitis) correctly was harder than classifying positive cases (i.e., with periodontitis) correctly. The effect size was quite large in absolute terms (i.e., a difference = 10%!), although a simple *z*-test indicates that this difference was not significant (*P* > 0.05).
- There was some evidence that augmentation led to improved results for these measures, although this was inconclusive here. However, one study by Mao used a different type of outcome measure that might have confounded results, i.e., any differences between augmented versus not augmented might have been due partially or wholly to differences in the different outcome measures. However, Mao (2023) study was removed from the analysis because it used a training sample to evaluate model performance instead of a testing or validation sample, indicating a high risk of bias. Therefore, except for sensitivity, all meta-analysis forest plots attached were carried out without data augmentation.
- For this studies considered here, data type (measures made on testing or training data) did not seem to affect the results much, although this was hampered by the fact that there were only a few studies where it appeared that quoted results were based on the training set only. Furthermore, a read through of the papers where data is split into testing, validation, and testing sets shows that measures within in each paper are lower for testing sets compared to results for training sets.
